# Physical Activity during Pregnancy and Childhood Obesity: Systematic Review and Meta-Analysis

**DOI:** 10.3390/jcm13133726

**Published:** 2024-06-26

**Authors:** Rubén Barakat, Cristina Silva-José, Miguel Sánchez-Polán, Dingfeng Zhang, Pablo Lobo, Gabriela De Roia, Rocío Montejo

**Affiliations:** 1 AFIPE Research Group, Faculty of Physical Activity and Sport Sciences—INEF, Universidad Politécnica de Madrid, 28040 Madrid, Spain; cristina.silva.jose@upm.es (C.S.-J.); dingfeng.zhang@alumnos.upm.es (D.Z.); 2 GICAF Research Group, Department of Education, Research and Evaluation Methods, Universidad Pontificia Comillas, 28049 Madrid, Spain; mspolan@comillas.edu; 3 Laboratorio de Estudios en Actividad Física (LEAF), Universidad de Flores (UFLO), Buenos Aires C1406, Argentina; pablo.lobo@uflouniversidad.edu.ar (P.L.); gabriela.deroia@uflouniversidad.edu.ar (G.D.R.); 4 Department of Obstetrics and Gynecology, Institute of Clinical Sciences, University of Gothenburg, 405 30 Gothenburg, Sweden; rocio.montejo.rodriguez@vgregion.se; 5 Department of Obstetrics and Gynecology, Sahlgrenska University Hospital, 413 45 Gothenburg, Sweden

**Keywords:** pregnancy, physical activity, exercise, childhood obesity

## Abstract

**Background and Objectives:** The repercussions of childhood overweight and obesity are multifaceted, extending beyond the realm of physiology and giving rise to psychological and emotional disturbances in affected children. The precise effects of gestational physical activity (PA) on parameters related to childhood overweight and obesity remain inadequately understood. The aim of this study (Registration CRD42022372490) was to evaluate the literature regarding the influence of PA during pregnancy on the risk of childhood overweight and obesity. **Materials and Methods:** Only randomized controlled trials (RCTs) were considered for inclusion. Determinant parameters of childhood obesity were analyzed. A total of 30 studies involving 16,137 pregnant women were examined. Five meta-analyses about the effects of PA during pregnancy on determinants of childhood overweight and obesity were conducted. **Results:** Although favorable trends were observed, Meta-Analyses showed no statistical differences in the effects of PA on weight at birth (Z = 0.03, *p* = 0.97), Ponderal Index at birth (Z = 0.64, *p* = 0.52), Macrosomia and Large for Gestational Age at birth (Z = 0.93, *p* = 0.35), children’s BMI (Z = 0.78, *p* = 0.44), weight (Z = 0.50, *p* = 0.62), and skinfold thicknesses (Z = 0.45, *p* = 0.65). **Conclusions:** The engagement in physical activity during pregnancy exhibits a favorable trend in parameters associated with childhood overweight and obesity while presenting no adverse effects on such outcomes.

## 1. Introduction

The growing percentage of children who are overweight and obese has emerged as a pervasive global concern, exhibiting a relentless upward trend that poses formidable challenges to mitigation efforts. Globally, in 2022, an estimated 37 million children under 5 years were classified as overweight. What was once perceived as primarily a concern in countries with high incomes has now become more prevalent in low- and middle-income territories. In Africa, the prevalence of overweight children under 5 years of age has surged by nearly 23% since 2000. Notably, nearly half of the children under 5 years of age who were overweight or obese in 2022 resided in Asia [1].

The repercussions of childhood overweight and obesity are multifaceted, extending beyond the realm of physiology and giving rise to psychological and emotional disturbances in affected children. Remarkably, chronic conditions that were historically prevalent only in adults are now increasingly diagnosed among children afflicted by obesity [2,3]. Such conditions encompass hypertension [4], type 2 diabetes, asthma [5], sleep apnea [6], joint disorders, and musculoskeletal pain [7], as well as gastrointestinal and hepatic complications [2,8,9]. Additionally, obesity can profoundly impact their psychological and emotional well-being, leading to conditions such as depression, diminished self-esteem [10,11,12], and body dissatisfaction [2,12].

Within the realm of potential causative factors, a substantial body of scientific literature grounded in an epigenetic framework delves into the genesis of this issue during the gestational period. It substantiates the notion of an unfavorable intrauterine environment, particularly in the metabolic context, as a paramount factor intricately linked to the burgeoning epidemic of childhood obesity. This assertion finds robust support in a plethora of studies dedicated to this subject matter [13,14,15,16].

This scientific evidence underscores the pivotal role of the gestational period in shaping the health of future populations. In this context, the repercussions of a healthy or imbalanced pregnancy extend far beyond the gestational period, affecting both the mother and the offspring in detectable ways [17].

Recent research indicates that the perinatal environment contributes to childhood obesity, irrespective of genetic predisposition and dietary habits [18,19]. Consequently, the pregnancy period emerges as a critical window for early prevention [20,21].

In this context, research suggests that fetal exposure to a metabolically adverse intrauterine environment is linked to subsequent complications in newborns. Indeed, specific studies even posit that genetic alterations in metabolic regulation during fetal life might impact the appetite, energy regulation, and metabolism of the newborn [22,23].

From a metabolic standpoint, a pivotal determinant for maintaining a healthy intrauterine environment is the significant aspect of appropriate maternal weight gain during pregnancy, which is closely associated with a woman’s pre-pregnancy BMI. The imbalance of these factors, coupled with their attendant complications, such as gestational diabetes (GD) and hypertension, is strongly correlated with an elevated risk of childhood obesity [24,25,26,27,28,29,30]. Current data indicate concerning trends. Approximately 34% of pregnant women exhibit a BMI exceeding 25 kg/m^2^ [31], although some studies suggest an even higher percentage nearing 50% [29,32]. Notably, 60% of pregnant women with obesity and 40% with normal weight surpass the recommendations established by the Institute of Medicine [33], amplifying the aforementioned risks.

In addition to the well-documented issue of fetal macrosomia [34,35], there exists a heightened risk of hypertension, pre-eclampsia [34,35,36], gestational diabetes [36,37,38], infections, thromboembolic disease [32], induced labor [32,36,38], and an increased likelihood of cesarean section [31,32,35,36,38,39], perinatal death [24,27,28,40], and maternal weight retention during the postpartum period [41].

Children born to mothers who are obese or overweight during pregnancy face elevated risks, including an increased likelihood of developing insulin resistance [42], requiring neonatal intensive care, being born preterm, having congenital anomalies, and needing treatment for hypoglycemia [32,36,38,41].

It is imperative to develop and implement strategies to manage maternal weight gain during pregnancy effectively, along with exploring other relevant epigenetic markers, to mitigate the escalating epidemic of childhood overweight and obesity. Achieving this goal necessitates a fundamental shift in maternal behaviors during pregnancy. The objective is to identify practical interventions that can substantially enhance the lifestyle of pregnant individuals, thereby fostering the well-being of future generations in the long term [43].

Engaging in physical activity during pregnancy, including exercises such as walking, swimming, yoga, and low-impact aerobics, offers numerous benefits. It is recommended that pregnant individuals accumulate moderate-intensity aerobic physical activity with a minimum of three days per week, although daily activity (avoiding a sedentary lifestyle) is encouraged to ensure consistent benefits and minimize the risk of injury [44].

In this context, over the last three decades, the practice of physical activity through pregnancy has emerged as a preventive factor against pre-, peri-, and postnatal complications, a fact substantiated by substantial scientific evidence. The recent recommendation by the World Health Organization [45], advocating for at least 150 min of moderate-intensity physical activity weekly for all healthy pregnant women to promote well-being, exemplifies this reality. However, the global data indicate a low prevalence of physical activity among pregnant women [46] which apparently could be directly associated with the increasing prevalence of childhood obesity.

The impact of prenatal physical activity on reducing childhood obesity rates has been well-documented. However, despite significant research supporting this trend, a broad and concrete consensus has not yet been established [15,17]. Rigorous review studies of the scientific literature on this topic are necessary to draw solid conclusions [14]. In this context, systematic reviews and meta-analyses of experimental studies, particularly those examining randomized controlled trials (RCTs), are considered the most reliable type of study. Given this context, the objective of this study was to systematically analyze the published literature regarding the influence of physical activity during pregnancy on childhood overweight and obesity risk.

## 2. Materials and Methods

A Systematic Review and Meta-Analysis (SR + MA) design was carried out based on the PRISMA guidelines [47].

Search strategy, database information, and data extraction were registered in PROSPERO, Registration No. CRD42022372490.

### 2.1. Eligibility Criteria

The PICOS framework was used to set the search strategy as registered in the protocol [47].

### 2.2. Population

The study population comprised healthy pregnant women without any contraindications to participate in a physical exercise intervention. Absolute contraindications were delineated as conditions to stop physical activity due to imminent risk for the mother or the fetus [44].

### 2.3. Intervention (Exposure)

Information on the characteristics of the intervention has been examined: type of intervention, supervision class, duration of class, and adherence of the participants to the intervention (Table 1).

### 2.4. Comparison

The comparator was defined as no exercise, which constituted the control group in the selected studies. Typically, this involved pregnant women undergoing regular follow-ups during pregnancy at health centers.

### 2.5. Outcome

The main outcome of the study was childhood obesity or directly related parameters and determinants of childhood obesity such as birth weight, newborn body mass index, macrosomia, Ponderal Index, or Large for Gestational Age (LGA). Relevant secondary variable outcomes were other parameters of the children (cardiovascular, motor, mental). Regarding birth weight, only studies that examined this parameter in relation to other childhood parameters were included.

### 2.6. Study Design

Only randomized controlled trials (RCTs) were considered for inclusion. Other types of study design were excluded from analysis in this review.

### 2.7. Data Sources

The initial search was performed by three different reviewers (DZ, MS-P, and CS) through Universidad Politécnica de Madrid software across several databases: EBSCO (involving five databases: Academic Search Premier, Education Resources Information Center, MEDLINE, SPORTDiscus, and OpenDissertations), Clinicaltrials.gov, Web of Science, Scopus, Cochrane Database of Systematic Reviews, and Physiotherapy Evidence Database (PEDro). Uniform article selection criteria were applied across listed databases to ensure consistency in the selection, accounting for differences in controlled vocabulary and selection syntax rules. Data Base Search Strategy was included as Supplementary Materials. The search terms employed were as follows:

- English: physical activity OR exercise OR training OR physical exercise OR fitness OR strength training OR physical intervention OR Pilates OR Yoga OR strengthening OR aerobic OR resistance training OR walking AND pregnancy OR maternal OR antenatal OR pregnant AND health OR wellbeing AND childhood obesity OR child follow-up OR infant adiposity OR paediatric obesity OR paediatric overweight OR macrosomic AND randomized clinical trial OR randomized controlled trial OR RCT.
- Spanish: actividad física OR ejercicio OR entrenamiento OR ejercicio físico OR fitness OR entrenamiento de fuerza OR intervención de actividad física OR Pilates OR Yoga OR fortalecimiento OR aeróbico OR entrenamiento de resistencia OR caminar AND embarazo OR materno OR antenatal OR embarazada AND salud OR bienestar AND obesidad infantil OR seguimiento infantil OR adiposidad infantil OR obesidad pediátrica OR sobrepeso pediátrico OR macrosomía AND ensayo clínico aleatorizado OR ensayo controlado aleatorizado OR ECA.

### 2.8. Study Selection and Data Extraction

In addition to RCTs, we conducted a complementary search for previously published systematic reviews in the same field to facilitate a comparative analysis of our results. RCTs published from 2010 to March 2024, in both languages, were evaluated, as we tried to focus on the latest scientific publications to update current knowledge on this topic.

Three researchers conducted an abstract/title search to identify exercise interventions during gestation. The main objective was to extract pertinent details to ensure that the resulting articles met the eligibility criteria, thus enabling the full-text reviews of these valid articles to be considered going forward.

After that, two researchers (GD and PL) screened the full-text files to evaluate the validity of the selected articles and to filter results of interest for data extraction. In cases where a study had multiple publications, we prioritized the most recent as the primary source.

In situations of one recommended article exclusion, both people endeavored to reach a consensus to have a final decision regarding its inclusion. In cases of persistent disagreement, a third person (RM) provided the opinion on whether the study should be included or excluded.

Data extraction was performed through an Excel spreadsheet. One researcher (MS-P) performed the extraction of the data, which was subsequently verified by a content expert to facilitate further analysis. A manual search of reference lists and relevant journals was performed to ensure a comprehensive review.

### 2.9. Statistical Analysis

Version 5.4 of the RevMan statistical package was used. Two distinct meta-analyses were conducted: one involving continuous variables, where the overall confidence interval (CI) was computed using the standardized mean difference, and another involving categorical variables (yes/no), where the number of events in each study group and its relative risk (RR) was documented. The total RR was calculated using a fixed effects model, accounting for the weight assigned to each study based on its sample size, thus establishing a compensated average [48].

In both analyses, the I^2^ statistic was employed to quantify the heterogeneity resulting from different interventions and study designs in each article. This statistic measures the variability in the effect of each intervention and is non-random. Heterogeneity was categorized as follows: low (25%), moderate (50%), and high (75%) [49].

### 2.10. Quality of Evidence and Risk of Bias Assessments

To assess the quality of analyzed evidence through selected articles, two reviewers (DZ and CS) utilized the GRADE framework in its website tool (GRADEpro) version [50]. Results of GRADE Assesment were included as Supplementary Materials. This tool, by inputting the results of performed analyses and the risk of bias of selected articles, allows researchers to determine the certainty of evidence and the importance of each outcome.

For evaluating the risk of bias (RoB) of analyzed articles, the RoB version 2 of the Cochrane Handbook was used, through RevMan software version 5.4. Potential sources of bias assessed included selection bias (including the generation of the sequence of randomization evaluation and how was the participant allocation), performance bias (compliance with the intervention for RCTs, i.e., blinding involving personnel or/and participants), detection bias (flawed outcome measurement, if exists outcome blinding), attrition bias (incomplete outcome data or a high rate of lost to follow-up), and reporting bias (selective or incomplete outcome reporting) [51].

To evaluate publication bias in each analysis, the Egger regression test was utilized due to its sensitivity in detecting publication bias in all included articles. Typically, significant publication bias is indicated when *p* < 0.1 [52].

**Table 1:** Characteristics of the analyzed references.

Author	Year	Country	N	EG	CG	Intervention. Physical Exercise Program							Main Variables Analyzed	Secondary Variables Analyzed
						Fq.	Intens.	Dur. of Program	Type of Intervent.	Superv. Class	Dur. of Class	Adh. %		
Bjøntegaard [53]	2021	Norway	281 BMI < 25	164	117	1	Mod	12 w	EP: Aerobic, strength, balance exercise	Yes	60 min	56.7	Childhood obesity At birth: birthweight (grams) 7 years: BMI, kg/m^2^	Daily activity of children
						2				No	45 min			
Braeken [54]	2020	Belgium	173 BMI ≥ 30	96	77	-	Lifestyle intervention	20 w	Nutrition advice physical activity,	Yes	-	-	Childhood anthropometrics At birth: birthweight 3 to 7 years: weight, BMI, Waist circumference Hip circumference Circumference at umbilicus level Ratios: waist-to-hip/waist-to-height	Neurocognitive development, eating habits and children cardiovascular
Chiavaroli [55]	2018	New Zealand	84 BMI 25–30	47	37	5	65% VO_2max_	16 w	Stationary cycling	No	40 min	-	Metabolism and body composition in mothers and offspring 1 year: waist circumference, hip circumference, waist-to-hip ratio, BMI 7 years: Total body fat (%), BMI	Maternal and neonatal outcomes
Clark [56]	2018	USA	36 BMI 18.5–35	14	22	3	Mod	20 w	EP: Aerobic	Yes	50 min	-	Neonatal body size At birth: weight, BMI, abdominal circumference, Ponderal Index	Neonatal outcomes
Dalrymple [57]	2020	UK	514 BMI ≥ 30	250	264	7	Light	8 w	Aerobic, nutrition advice	No	-	-	Childhood adiposity and cardiovascular function At birth: Birthweight, Birthweight > 4 kg, Large for Gestational Age (LGA) > 90th Centile Subscapular skinfold thickness Triceps skinfold thickness 3 years: Weight, Different skinfold thickness, Waist Circumference, Mid upper arm circumference, BMI for age Z-Score	Neonatal and infant outcomes
Dodd [58]	2014a	Australia	2202 BMI ≥ 25	1105	1097	-	Lifestyle intervention	30 w	Walking, nutrition advice	No	-	-	Infant outcomes At birth: LGA, Birth weight above 4000 g	Maternal outcomes
Dodd [59]	2014b	Australia	2142 BMI ≥ 25	1075	1067	-	Lifestyle intervention	30 w	Walking, nutrition advice	No	-	-	Infant outcomes At birth: Birth weight, Birth weight (Z-Score), Birth weight ≥ 4.5 kg, Ponderal Index	Maternal outcomes
Dodd [60]	2018	Australia	2136 BMI ≥ 25	1071	1065	-	Lifestyle intervention	30 w	Walking, nutrition advice	No	-	-	Children anthropometry 1.5 years: Weight, BMI Z-score > 85th, BMI Z score, mean (SD, Abdomen circumference, BMI Z-Score > 90th, Bio-impedance	Children dietary intake and family food behaviour
Dodd [61]	2020	Australia	1418 BMI ≥ 25	727	691	-	Counselling	20 w	Walking, nutrition advice	No	-	77.2	Childhood obesity 3 to 5 years: Weight, BMI, BMI Z-Score, BMI Z-Score > 85th percentile, BMI Z-Score > 90th percentile, Weight/height ratio Weight/length ratio Z-Score, Abdomen circumference	Infant outcomes
Gallagher [62]	2018	USA	196 BMI ≥ 25	97	99	-	Lifestyle intervention	22 w	Physical activity, nutrition advice	No	-	34.2	Infant fat free mass At birth: LGA (>90th percentile), Birth weight, Weight-for-age Z-Score 2 to 4 days after birth: Percentage fat, Total fat mass, Ponderal Index, Sum of skinfolds	Infant outcomes
Hopkins [63]	2010	New Zealand	84 BMI ≥ 25	47	37	5	65% VO_2max_	16 w	Stationary cycling	No	40 min	-	Maternal insulin sensitivity At birth: Birthweight, BMI, Ponderal Index. 17 days: Body weight, Fat mass.	Neonatal outcomes
Huang [64]	2019	Australia	42 BMI ≥ 20	23	19	-	Lifestyle intervention	12 w	Nutrition advice, walking	No	-	87.2	Gestational weight gain At birth: Birthweight, Ponderal Index, body fat, Body fat mass. 3 months: Ponderal Index, weight.	Infant outcomes
Kolu [65]	2016	Finland	173 BMI ≥ 25	85	88	3	Lifestyle interv. Mod	28 w	Nutrition, physical activity advice	No	30 min	-	Type 2 diabetes mellitus, gestational weight gain At 7 years: BMI	Infant outcomes
Kong [66]	2014	USA	34 BMI ≥ 25	15	19	5	Mod	20 w	Walking	No	30 min	-	Post-partum weight retention 1 month: bodyweight weight (Z-Score), weight-for-length (Z-Score), Fat mass 6 months: Bodyweight, Weight (Z-Score), weight-for-length (Z-Score), Fat mass	Children anthropometry
Luoto [67]	2011	Finland	399 BMI ≥ 25	219	180	4 times	-	29 wk	Nutrition, physical activity advice	-	-	-	Gestational diabetes and birthweight At birth: Birthweight, LGA Macrosomia (birthweight > 4500 g) Macrosomia (birthweight > 4000 g) Ponderal Index	Neotanal and child outcomes
May [68]	2023	USA	56 BMI 18.5–40	31	25	3	Mod	24 wk	EP: Aerobic exercises	Yes	50 min	80	Infant cardiac function and outflow 1 month: Weight, BMI	Infant outcomes
McMillan [69]	2019	USA	60 BMI 18.5–35	33	27	3	Mod	20 w	EP: Aerobic exercises	Yes	50 min	-	Infant Neuromotor Development 1 month: BMI, Weight	Infant outcomes
Mustila [70]	2012	Finland	72 BMI ≥ 25	34	38	1	Lifestyle intervention	28 w	Nutrition, physical activity advice	Yes	-	-	Offspring Weight Gain At birth: Birthweight, Small for Gestational Age (SGA), LGA, Macrosomia. 0–48 months: Weight-for-length/height (Z-Score) 24 to 48 months: BMI (Z-Score)	Infant outcomes
Mustila [71]	2013	Finland	185 BMI ≥ 25	96	89	5	Counselling Light	22 w	Aerobic and strength exercise, nutrition advice	No	30 min	-	Childhood obesity at birth: Birthweight, Ponderal Index, SGA, LGA, BMI. 4 months: BMI, Weight-for-length 6 months: BMI, Weight-for-length. 12 months: BMI, Weight-for-length.	Neonatal and infant outcomes
Mustila [72]	2018	Finland	147 BMI ≥ 25	71	76	-	Counselling	13 w	Walking and nutrition advice	No	-	-	Offspring’s weight gain at birth: Birthweight, LGA. 6 years: BMI > 25 kg/m^2^, BMI >30 kg/m^2^, Weight-for-length ≥ 10% Weight-for-length > 20%	Maternal and neonatal outcomes
Patel [73]	2017	UK	698 BMI ≥ 30	342	356	-	Behavioural intervention	18 w	Nutrition, physical activity advice	-	-	47.3	Childhood adiposity At birth: Birthweight, LGA, 6 months: Different skinfold thickness Abdominal circumference (cm) BMI for age (Z-Score)	Maternal dietary and physical activity
Perales [74]	2020	Spain	BMI 18.5–30	688	660	3	Mod	28 w	EP: Aerobic, resistance, pelvic floor training	Yes	50–55 min	95	Maternal cardio-metabolic health at birth: Birthweight, Low birthweight Macrosomia 1 year: BMI, Overweight/obesity 6 years: Overweight/obesity	Maternal/offspring health outcomes
Phelan [75]	2019	USA	835 BMI ≥ 25	423	412	-	Lifestyle intervention	20 w	Nutrition, physical activity advice	No	-	80	Post-partum weight retention At birth: Weight for length (Z-Score) Different skinfold thickness 1 year: Weight for length (Z-Score) Different skinfold thickness	Children anthropometry
Poston [76]	2015	UK	1555 BMI ≥ 30	783	772	1	Behavioural intervention	8 w	Nutrition advice	Yes	60 min	-	Gestacional diabetes and LGA At birth: Birthweight, Birthweight > 4 kg LGA (customised birthweight centiles) ≥90th Population birthweight centiles ≥ 90th	Infant outcomes
Rauh [77]	2015	Germany	250 BMI ≥ 18.5	167	83	2 total	Lifestyle intervention	18 w	Nutrition, physical activity advice	Yes	-	-	Post-partum weight retention 3 days to 12 months: Weight	Infant weight outcomes
Ronnberg [78]	2017	Sweden	374 Healthy BMI	192	182	-	Lifestyle intervention	-	Nutrition advice	-	-	-	Childhood obesity At birth: Birthweight, BMI, BMI (Z-Score) LGA, SGA, Ponderal Index 5 years: BMI, BMI (Z-Score)	Risk estimates for offspring obesity in relation to maternal GWG
Sandborg [79]	2022	Sweden	247 Healthy BMI	122	125	-	Lifestyle intervention	22 w	APP (nutrition, exercise advice and feedback)	-	-	-	Infant body composition At birth: Birthweight 1 to 2 weeks: Weight, BMI, Body fat, Fat mass index kg/m^2^	Infant outcomes
Tanvig [80]	2014	Denmark	157 BMI 30–45	82	75	7	Lifestyle intervention Mod	22 w	Aerobic exercise	No	30–60 min	52	Offspring anthropometrics and body Composition At birth: Birthweight, Birthweight (Z-Score), Macrosomia, LGA, Abdominal circumference (cm) 2.8 years: BMI, overweight or obese, BMI (Z-Score), Weight, Abdominal circumference, Triceps/Subscapular skinfold (mm)	Neonatal and infant outcomes
Tanvig [81]	2015	Denmark	150 BMI 30–45	77	73	7	Lifestyle intervention Mod	22 w	Aerobic exercise	No	30–60 min	52	Offspring metabolic risk factor at birth: Birthweight, Abdominal circumference 0 to 12 months: Change in weight 2.8 years: BMI Z-Score, overweight or obese, Abdominal circumference	Infant outcomes
Vesco [82]	2016	USA	89 BMI ≥ 30	43	46	-	Counselling	20 w	Nutrition advice	No	-	-	Post-partum weight retention At birth: Birtheight, Weight for age (Z-Score) 2 weeks: Weight, Weight for age (Z-Score) Sum of triceps + subscapular skinfold thicknesses (mm) 1 year: Weight, Weight for age (Z-Score) Weight for length (Z-Score) Sum of triceps + subscapular skinfold thicknesses (mm)	Infant body composition

Author: study reference. Year: publication year. N: total number of participants. EG: number of participants in the intervention group. CG: number of participants in the control group. Fq.: frequency in a week of exercise sessions. Intens.: intensity. Dur. of prog.: program duration in weeks. Type of intervent.: type of intervention: exercise program (EP: aerobic, muscle strengthening, etc.) or physical activity and nutritional advice. Superv. Class: whether or not intervention was supervised. Dur. of class: minutes of each session. Adh.: adherence (compliance) to the intervention (%).

## 3. Results

### 3.1. Study Selection

**Figure 1:**
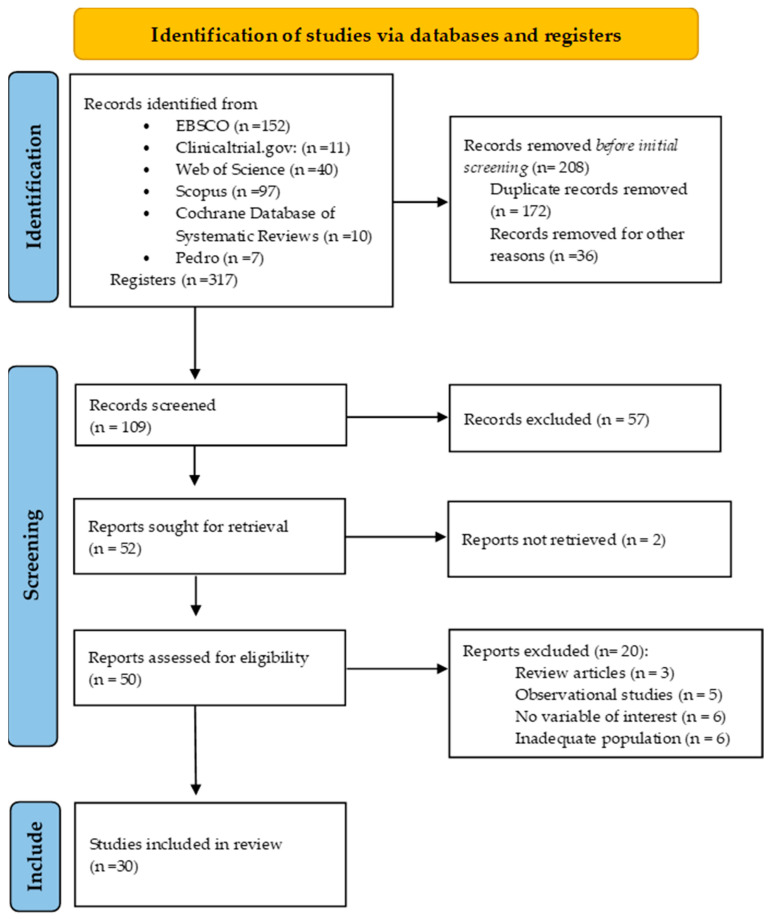
Flow diagram of the search.

A total of 317 references were initially evaluated during the first search, thus removing 208 before screening: duplicate records (n = 172) and other reasons (n = 36). A total of 59 articles were excluded due to not meeting inclusion criteria (n = 57) and reports not retrieved (n = 2). Subsequently, 20 articles were not included due to the following reasons: being a review article (n = 3) or observational study (n = 5), no variable of interest (n = 6), or inadequate population (n = 6). The detailed study selection process is illustrated in Figure 1.

Ultimately, 30 studies were identified that met the inclusion criteria, with 16,137 women from 11 countries and three continents. All studies were RCTs, and the intervention used was supervised physical exercise [53,56,68,69,74] or advice and recommendations [54,55,57,58,59,60,61,62,63,64,65,66,67,70,71,72,73,75,76,77,78,79,80,81,82] (Table 1).

### 3.2. Risk of Bias Assessment

The collective certainty of the evidence was established as “high” and the importance of each outcome was established as “important”. Regarding RoB analysis, it was observed that blinding of participants to each group is typically unfeasible due to the characteristics of the physical activity intervention, resulting in unclear or high risk of bias depending on how it was reported. In certain instances, other sources of bias included the inability to access the published article protocol (for comparing planned and measured outcomes) and insufficient transparency in reporting the randomization. Overall, the studies exhibited a low risk of bias across the types assessed. The risk of bias analysis is depicted individually in each of the following meta-analyses.

### 3.3. PA Effect during Gestation on Birthweight

**Figure 2:**
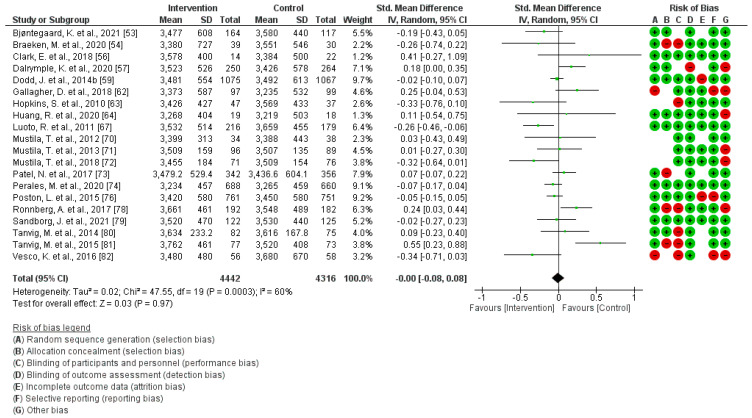
Effect of PA during pregnancy on birthweight [53,54,56,57,59,62,63,64,67,70,71,72,73,74,76,78,79,80,81,82].

A total of 20 studies were incorporated into this analysis [53,54,56,57,59,62,63,64,67,70,71,72,73,74,76,78,79,80,81,82]. Regular physical activity during pregnancy did not show a relationship with weight at birth (Z = 0.03; *p* = 0.97) (Std. Mean Dif., Random, 95% CI = −0.00 [−0.08, 0.08] I^2^ = 60%, P*_heterogeneity_* = 0.0003). The graphic corresponding to the current Meta-Analysis is illustrated in Figure 2. Publication bias assessment showed no potential publication bias (*p* = 0.93) in this analysis.

### 3.4. PA Effect during Pregnancy on Ponderal Index at Birth

**Figure 3:**
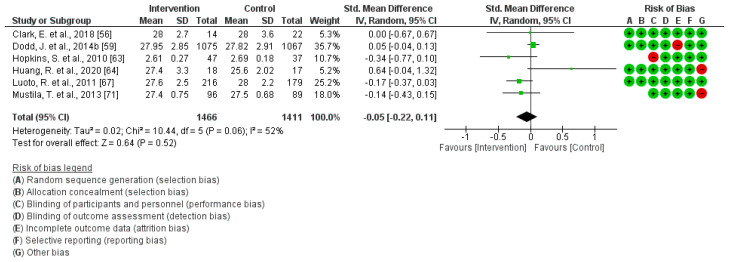
Effect of PA during pregnancy on Ponderal Index at birth [56,59,64,67,71].

A total of six studies were included in this analysis [56,59,64,67,71]. The findings indicate that engaging in regular exercise during pregnancy does not exhibit a significant association with Ponderal Index at birth (Z = 0.64; *p* = 0.52) (Standardized Mean Difference, Random Effects Model, 95% Confidence Interval = −0.05 (−0.22, 0.11), I^2^ = 52%, P*_heterogeneity_* = 0.06). Figure 3 shows the corresponding graphic of this Meta-Analysis. The evaluation of publication bias across the reviewed articles indicated minimal evidence of bias, with a *p*-value of 0.48.

### 3.5. Effect of PA during Pregnancy on Macrosomia and Large for Gestational Age at Birth

**Figure 4:**
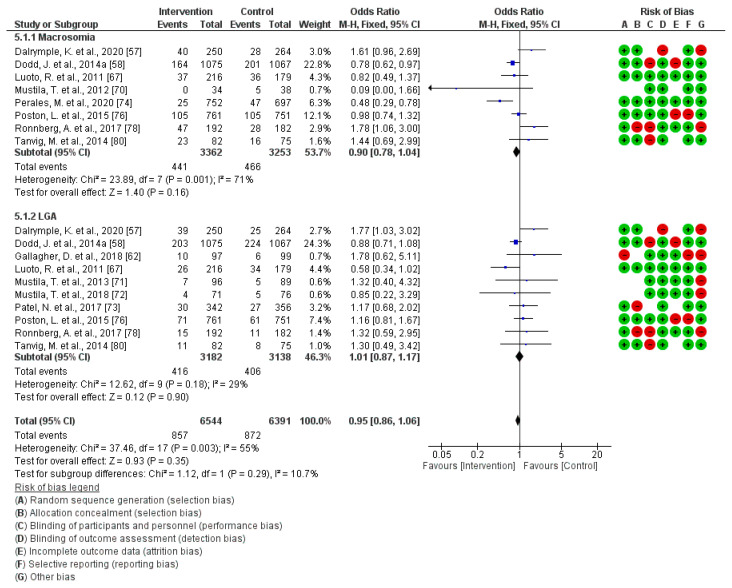
Effect of PA during pregnancy on Macrosomia and Large for Gestational Age at birth [57,58,62,67,70,71,72,73,74,76,78,80].

This analysis included a total of 12 studies [57,58,62,67,70,71,72,73,74,76,78,80]. There was no significant association (Z = 0.93; *p* = 0.35) observed between exercise during gestation and the likelihood of Macrosomia and Large for Gestational Age at birth (Relative Risk = 0.95, 95% Confidence Interval = 0.86, 1.06, I^2^ = 55%, P*_heterogeneity_* = 0.003). Figure 4 illustrates the corresponding graphic for the current meta-analysis. The assessment of bias in publication across the analyzed articles revealed negligible evidence of bias, as reflected by a *p*-value of 0.09. 

### 3.6. Effect of PA during Pregnancy on Children’s BMI (1 Month–7 Years)

**Figure 5:**
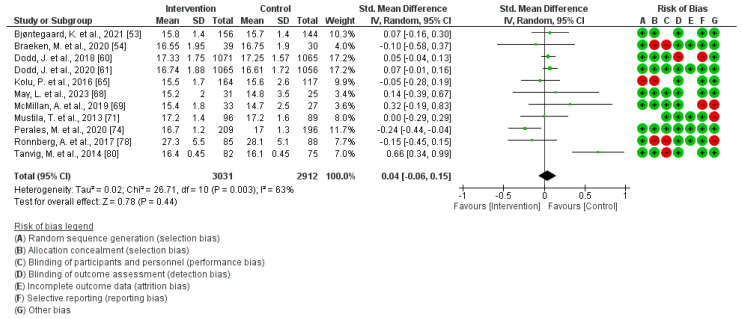
PA effect during pregnancy on children´s BMI [53,54,60,61,65,68,69,71,74,78,80].

A total of 11 studies were included in this analysis [53,54,60,61,65,68,69,71,74,78,80]. The findings indicate that engaging in a physical activity program across pregnancy does not exhibit a significant association with children´s BMI (Z = 0.78; *p* = 0.44) (Standardized Mean Difference, Random Effects Model, 95% Confidence Interval = 0.04 (−0.06, 0.15), I^2^ = 63%, P*_heterogeneity_* = 0.003). Figure 5 illustrates the corresponding plot of the Meta-Analysis. Analysis of the publication bias risk in the surveyed articles showed no significant evidence of bias, evidenced by a *p*-value of 0.78.

### 3.7. Effect of PA during Pregnancy on Children´s Weight (1 Month–7 Years)

**Figure 6:**
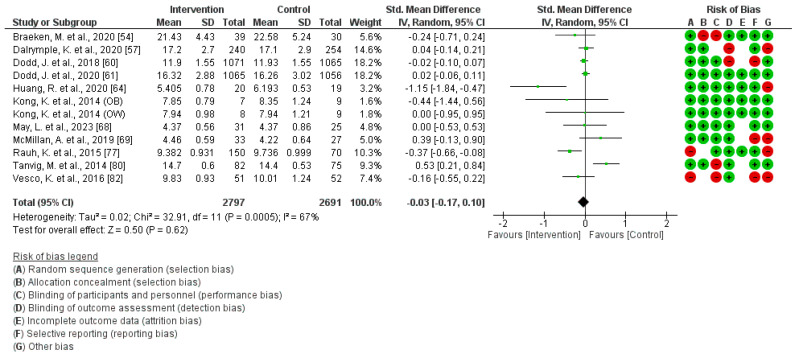
Effect of PA during pregnancy on children´s weight [54,57,60,61,64,66,68,69,77,80,82].

A total of 11 studies were included in this analysis [54,57,60,61,64,66,68,69,77,80,82]. The findings indicate there was no significant association between physical activity and children´s weight (Z = 0.50; *p* = 0.62) (Standardized Mean Difference, Random Effects Model, 95% Confidence Interval = −0.03 (−0.17, 0.10), I^2^ = 67%, P*_heterogeneity_* = 0.0005). Figure 6 illustrates the corresponding forest plot of the Meta-Analysis. The examination of potential publication bias within the studied articles found scant evidence of bias, as demonstrated by a *p*-value of 0.58.

### 3.8. Effect of PA during Pregnancy on Children´s Skinfold Thicknesses (Abdominal/Triceps/Subescapularis)

**Figure 7:**
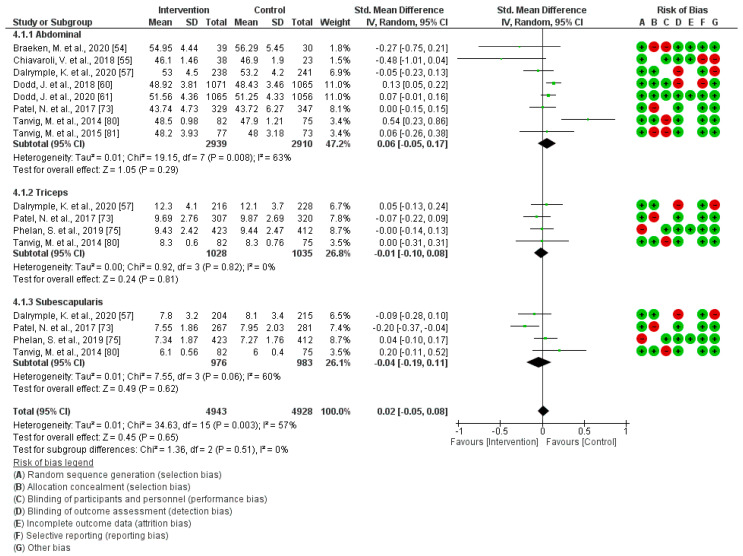
Effect of PA during pregnancy on children´s skinfold thicknesses [54,55,57,60,61,73,75,80,81].

A total of nine studies were included in this analysis [54,55,57,60,61,73,75,80,81]. The results indicate that regular exercise during pregnancy does not exhibit a significant association with children´s skinfold thicknesses (Z = 0.45; *p* = 0.65) (Standardized Mean Difference, Random Effects Model, 95% Confidence Interval = 0.02 (−0.05, 0.08), I^2^ = 57%, P*_heterogeneity_* = 0.003). Figure 7 illustrates the graphic of the analysis. The assessment of publication bias risk across the analyzed articles revealed no notable evidence of publication bias (*p* = 0.41) in this analysis.

## 4. Discussion

Our study aimed to discern the impact of physical activity during pregnancy on childhood obesity indicators, such as birthweight, BMI, skinfold thickness, and the incidence of macrosomia and LGA outcomes. To the best of our knowledge, this represents the first SR + MA conducted on this particular scientific subject.

The absence of notable differences in birthweight and BMI among children born to exercising mothers during pregnancy invites contemplation of underlying biological and physiological mechanisms. Exercise may modulate maternal energy balance without disrupting fetal growth parameters, possibly through adaptive responses that preserve fetal nourishment. This equilibrium potentially reflects evolved maternal capacities to support fetal development despite varied energy expenditures. Further exploration into how maternal exercise alters gestational resource allocation may illuminate the subtleties of this relationship.

The present meta-analysis indicates that maternal physical activity during pregnancy does not result in a significant difference in fetal birthweight compared to non-exercising controls, further supporting the growing consensus that maternal exercise is a safe practice for fetal development.

This lack of significant impact on fetal weight may be attributed to the adaptive capabilities of the placenta to regulate fetal nutrition and growth, as hypothesized by various researchers [83]. It is plausible that exercise-induced enhancements in placental function may offset potential changes in fetal growth parameters [84]. Moreover, the homeostatic mechanisms of the mother’s body during pregnancy are known to be highly efficient in maintaining energy balance. Even vigorous exercise in active women showed reassuring fetal Doppler indices, heart tracings, and biophysical profiles post-exercise, supporting the safety of continuing or initiating moderate to vigorous exercise during pregnancy [85].

Nevertheless, the substantial heterogeneity observed (I^2^ = 60%) suggests variability among the included studies, which could be due to differences in the type, frequency, duration, and intensity of the exercise regimes prescribed. Additionally, the potential impact of maternal nutrition and pre-pregnancy BMI on birthweight outcomes cannot be overlooked as these factors have been shown to have a significant influence on fetal growth [86].

Our findings suggest that engagement in physical activity during pregnancy does not significantly affect the likelihood of macrosomia. The pooled odds ratio (OR) for macrosomia across studies was close to 1, suggesting no substantial difference between the intervention (exercise) and control groups. This aligns with previous research suggesting that moderate exercise during pregnancy is not associated with an increased risk of delivering a macrosomic infant [59,67]. Similarly, the incidence of LGA infants did not significantly differ between the two groups. These results are particularly reassuring given the concerns regarding the potential for maternal exercise to alter fetal growth patterns.

In examining the longer-term effects of prenatal exercise, our analysis did not reveal significant differences in BMI during childhood (ages 5–7 years) or in BMI at birth. These findings contribute to the growing body of evidence suggesting that prenatal physical activity does not adversely affect child growth trajectories as measured by BMI.

Moreover, the standard mean differences (SMD) for birth weight and BMI indices in infancy and early childhood were negligible, further underscoring the safety profile of prenatal exercise with regard to these outcomes. The heterogeneity observed in some of the analyses was moderate, indicating that, while there may be variations across individual studies, the overall trend suggests no adverse impact.

The results of this meta-analysis also support current recommendations encouraging pregnant women to engage in regular, moderate-intensity physical activity, as it does not seem to compromise neonatal weight or childhood development, which are important predictors of health in later life. This is consistent with guidelines from health authorities that advocate for the benefits of physical activity during pregnancy for both maternal and fetal health [44,47].

Previous studies have offered varying results, some suggesting that exercise during pregnancy may reduce the risk of obesity in offspring, while others observed no effect. Our results align with the latter, adding to the narrative that exercise is a nuanced factor in the gestational period. Discrepancies across studies could be attributed to variability in exercise regimes, intensity, and duration, as well as methodological differences, such as the timing of outcome measurement and the specific populations studied.

The burgeoning prevalence of obesity in pregnant women and its ramifications not just for maternal health but for the lifelong health of offspring demand multifaceted intervention strategies.

The synthesis of these findings spotlights a critical intervention point: the promotion of exercise during pregnancy. Exercise has been touted for its broad spectrum of health benefits, including weight management, improved cardiovascular health, and enhanced mood and energy levels. By extrapolation, engaging in regular physical activity during pregnancy could hypothetically counteract the factors contributing to the development of childhood obesity. For instance, if maternal obesity can induce alterations in gut microbiota that predispose children to obesity, as outlined by Isolauri et al. (2016) [16], then the potential of exercise to improve gut health and reduce inflammation could be a key to breaking this cycle.

Exercise during pregnancy might also mitigate the risk factors outlined by Fraser et al. (2010) [28], who report that maternal weight gain is associated with obesity and adverse metabolic traits in childhood. By potentially moderating gestational weight gain, exercise could reduce the risks of delivering LGA infants, a concern raised in the consensus by Poston et al. (2011) [30], which also notes the lifelong health impact on the child due to maternal obesity.

Furthermore, considering Athukorala et al.’s (2010) findings [29], which connect adverse pregnancy outcomes with maternal overweight and obesity, exercise might serve as a preventive measure against such outcomes. Additionally, it could alleviate some of the gestational diabetes risks that Gillman et al. (2003) [26] indicate as precursors to adolescent obesity.

Therefore, promoting physical activity during pregnancy could be a strategic public health recommendation. Such an approach aligns with the view that preventive strategies focusing on maternal health may offer a viable route to curb the transgenerational perpetuation of obesity. While further research is required to establish a definitive causal link, the collective evidence points toward exercise during pregnancy as a promising intervention for improving both maternal and child health outcomes.

In our study, we leveraged a robust meta-analytic methodology which allowed us to integrate data across multiple studies enhancing the statistical power and reliability of our findings due to the large, combined sample size. The comprehensive nature of our meta-analysis, encompassing a broad spectrum of exercise types, intensities, and durations during pregnancy, contributes to a more generalized understanding of the effects of exercise on neonatal outcomes.

However, there are several limitations that must be acknowledged. The heterogeneity observed among the included studies is noteworthy. Variability in exercise regimens—ranging from type and intensity to duration—can significantly affect the comparability of the outcomes. This heterogeneity might limit the specificity of our conclusions regarding which exercise parameters are most beneficial during pregnancy. Furthermore, the quality of data available from the included studies is a concern, especially when considering that many relied on self-reported exercise, which is susceptible to recall bias. This method of data collection may not accurately capture the true exercise behaviors of the participants and could skew the associations observed.

These limitations should be carefully considered when interpreting the results. While our analysis suggests certain trends and associations, the impact of the aforementioned factors on our conclusions cannot be overlooked. Future research would benefit from more standardized protocols for exercise interventions during pregnancy and more objective measures of physical activity to reduce the risk of bias and improve the accuracy of findings.

Future research should aim to establish standardized exercise protocols during pregnancy to better quantify the relationship between maternal exercise and neonatal outcomes. Moreover, long-term follow-up studies that extend beyond early childhood could provide further insights into the potential impact of maternal exercise on offspring health.

Our findings indicate that physical activity during pregnancy did not significantly alter the birthweight or BMI during the early years of life, which is consistent with prior research suggesting that gestational exercise is beneficial for maternal health without adversely affecting neonatal outcomes. These results may be explained by the hypothesis that physical activity optimizes maternal energy balance and fosters an environment conducive to healthy fetal development. Exercise might trigger adaptive mechanisms that safeguard the fetus, maintaining nutrient delivery even amidst increased maternal energy expenditure.

Finally, while physical activity offers numerous benefits during pregnancy, it is important to consider potential risks, such as musculoskeletal injuries and falls, which could adversely affect the pregnancy. Appropriate exercise modifications and precautions can help mitigate these risks. Additionally, maternal age plays a significant role in pregnancy outcomes, with older mothers potentially facing higher risks of complications. Therefore, tailored exercise recommendations based on maternal age and fitness levels are crucial for optimizing both maternal and fetal health.

## 5. Conclusions

The present study found no statistical differences in the effects of physical activity on birth weight, Ponderal Index at birth, Macrosomia, LGA, children’s BMI, weight, and skinfold thicknesses. In conclusion, our comprehensive analysis adds to the evidence base indicating that engagement in physical activity during pregnancy demonstrates a favorable trend in parameters associated with childhood overweight and obesity while posing no discernible adverse effects. These findings should reassure health professionals and pregnant women about the safety of maintaining an active lifestyle during pregnancy.

## Supplementary Materials

The following supporting information can be downloaded at: https://www.mdpi.com/article/10.3390/jcm13133726/s1, Results of GRADE Assesment and Data Base Search Strategy.
