# Supplementary material for: Physical Activity during Pregnancy and Childhood Obesity: Systematic Review and Meta-Analysis
Source: J Clin Med. 2024 Jun 26;13(13):3726. doi: 10.3390/jcm13133726 (PMC11242316; doi:10.3390/jcm13133726)
Supplement: Supplementary file 1 [file jcm-13-03726-s001.zip › jcm-3003648-supplementary.pdf]

Database search strategy

EBSCO English

(“physical activity” OR “exercise” OR “training” OR “physical exercise” OR “fitness” OR “strength training” OR “physical intervention” OR “Pilates” OR “Yoga” OR “strengthening” OR “aerobic” OR “resistance training” OR “walking”) AND (“pregnancy” OR “maternal” OR “antenatal” OR “pregnant”) AND (“health” OR “wellbeing”) AND (“childhood obesity” OR “child follow-up” OR “infant adiposity” OR “paediatric obesity” OR “paediatric overweight” OR “macrosomia”) AND (“randomized clinical trial” OR “randomized controlled trial” OR “RCT”).

EBSCO Spanish

(“actividad física” OR “ejercicio” OR “entrenamiento” OR “ejercicio físico” OR “fitness” OR “entrenamiento de fuerza” OR “intervención de actividad física” OR “Pilates” OR “Yoga” OR “fortalecimiento” OR “aeróbico” OR “entrenamiento de resistencia” OR “caminar”) AND (“embarazo” OR “materno” OR “antenatal” OR “embarazada”) AND (“salud” OR “bienestar”) AND (“obesidad infantil” OR “seguimiento infantil” OR “adiposidad infantil” OR “obesidad pediátrica” OR “sobrepeso pediátrico” OR “macrosomía”) AND (“ensayo clínico aleatorizado” OR “ensayo controlado aleatorizado” OR “ECA”).

Clinicaltrials.gov English

(“childhood obesity” OR “child follow-up” OR “infant adiposity” OR “paediatric obesity” OR “paediatric overweight” OR “macrosomia”) AND (“physical activity” OR “exercise” OR “training” OR “physical exercise” OR “fitness” OR “strength training” OR “physical intervention” OR “Pilates” OR “Yoga” OR “strengthening” OR “aerobic” OR “resistance training” OR “walking”).

Clinicaltrials.gov Spanish

(“obesidad infantil” OR “seguimiento infantil” OR “adiposidad infantil” OR “obesidad pediátrica” OR “sobrepeso pediátrico” OR “macrosomía”) AND (“actividad física” OR “ejercicio” OR “entrenamiento” OR “ejercicio físico” OR “fitness” OR “entrenamiento de fuerza” OR “intervención de actividad física” OR “Pilates” OR “Yoga” OR “fortalecimiento” OR “aeróbico” OR “entrenamiento de resistencia” OR “caminar”).

Web of Science English

(“physical activity” OR “exercise” OR “training” OR “physical exercise” OR “fitness” OR “strength training” OR “physical intervention” OR “Pilates” OR “Yoga” OR “strengthening” OR “aerobic” OR “resistance training” OR “walking”) AND (“pregnancy” OR “maternal” OR “antenatal” OR “pregnant”) AND (“health” OR “wellbeing”) AND (“childhood obesity” OR “child follow-up” OR “infant adiposity” OR “paediatric obesity” OR “paediatric overweight” OR “macrosomia”) AND (“randomized clinical trial” OR “randomized controlled trial” OR “RCT”).

Web of Science Spanish

(“actividad física” OR “ejercicio” OR “entrenamiento” OR “ejercicio físico” OR “fitness” OR “entrenamiento de fuerza” OR “intervención de actividad física” OR “Pilates” OR “Yoga” OR “fortalecimiento” OR “aeróbico” OR “entrenamiento de resistencia” OR “caminar”) AND (“embarazo” OR “materno” OR “antenatal” OR “embarazada”) AND (“salud” OR “bienestar”) AND (“obesidad infantil” OR “seguimiento infantil” OR “adiposidad infantil” OR “obesidad pediátrica” OR “sobrepeso pediátrico” OR “macrosomía”) AND (“ensayo clínico aleatorizado” OR “ensayo controlado aleatorizado” OR “ECA”).

Scopus English

(“physical” AND “activity”) OR (“exercise”) OR (“training”) OR (“physical” AND “exercise”) OR (“fitness”) OR (“strength” AND “training”) OR (“physical” AND “intervention”) OR (“Pilates”) OR (“Yoga”) OR (“strengthening”) OR (“aerobic”) OR (“resistance” AND “training”) OR (“walking”) AND (“pregnancy”) OR (“maternal”) OR (“antenatal”) OR (“pregnant”) AND (“health”) OR (“wellbeing”) AND (“childhood” AND “obesity”) OR (“child” AND “follow-up”) OR (“infant” AND “adiposity”) OR (“paediatric” AND “obesity”) OR (“paediatric” AND “overweight”) OR (“macrosomia”) AND (“randomized” AND “clinical” AND “trial”) OR “randomized” AND “controlled” AND “trial”) OR (“RCT”).

Scopus Spanish

(“actividad” AND “física”) OR (“ejercicio”) OR (“entrenamiento”) OR (“ejercicio” AND “físico”) OR (“fitness”) OR (“entrenamiento” AND “de” AND “fuerza”) OR (“intervención” AND “de” AND “actividad” AND “física”) OR (“Pilates”) OR (“Yoga”) OR (“fortalecimiento”) OR (“aeróbico”) OR (“entrenamiento” AND “de” AND “resistencia”) OR (“caminar”) AND (“embarazo”) OR (“materno”) OR (“antenatal”) OR (“embarazada”) AND (“salud”) OR (“bienestar”) AND (“obesidad” AND “infantil”) OR (“seguimiento” AND “infantil”) OR (“adiposidad” AND “infantil”) OR (“obesidad” AND “pediátrica”) OR (“sobrepeso” AND “pediátrico”) OR (“macrosomía”) AND (“ensayo” AND “clínico” AND “aleatorizado”) OR (“ensayo” AND “controlado” AND “aleatorizado”) OR (“ECA”).

PEDRO English

Abstract & Title (“childhood obesity*”) AND (“pregnancy*”) AND Therapy (“fitness training”) AND Method (“clinical trial”).

Abstract & Title (“macrosomia*”) AND (“pregnancy*”) AND Therapy (“fitness training”) AND Method (“clinical trial”).

PEDRO Spanish

Abstract & Title (“obesidad infantil*”) AND (“embarazo*”) AND Therapy (“fitness training”) AND Method (“clinical trial”).

Abstract & Title (“macrosomia*”) AND (“embarazo*”) AND Therapy (“fitness training”) AND Method (“clinical trial”).

Cochrane Database of Systematic Reviews English

(“physical activity” OR “exercise” OR “training” OR “physical exercise” OR “fitness” OR “strength training” OR “physical intervention” OR “Pilates” OR “Yoga” OR “strengthening” OR “aerobic” OR “resistance training” OR “walking”) AND (“pregnancy” OR “maternal” OR “antenatal” OR “pregnant”) AND (“health” OR “wellbeing”) AND (“childhood obesity” OR “child follow-up” OR “infant adiposity” OR “paediatric obesity” OR “paediatric overweight” OR “macrosomia”) AND (“randomized clinical trial” OR “randomized controlled trial” OR “RCT”).

Cochrane Database of Systematic Reviews Spanish

(“actividad física” OR “ejercicio” OR “entrenamiento” OR “ejercicio físico” OR “fitness” OR “entrenamiento de fuerza” OR “intervención de actividad física” OR “Pilates” OR “Yoga” OR “fortalecimiento” OR “aeróbico” OR “entrenamiento de resistencia” OR “caminar”) AND (“embarazo” OR “materno” OR “antenatal” OR “embarazada”) AND (“salud” OR “bienestar”) AND (“obesidad infantil” OR “seguimiento infantil” OR “adiposidad infantil” OR “obesidad

pediátrica" OR "sobrepeso pediátrico" OR "macrosomía") AND ("ensayo clínico aleatorizado" OR "ensayo controlado aleatorizado" OR "ECA").
